# The A, C, G, and T of Genome Assembly

**DOI:** 10.1155/2016/6329217

**Published:** 2016-05-10

**Authors:** Bilal Wajid, Muhammad U. Sohail, Ali R. Ekti, Erchin Serpedin

**Affiliations:** ^1^Department of EE, University of Engineering & Technology, Lahore, Punjab 54890, Pakistan; ^2^Department of Physiology, Government College University, Faisalabad, Punjab 38000, Pakistan; ^3^Department of ECE, Gannon University, Erie, PA 16501, USA; ^4^Department of ECE, Texas A&M University, College Station, TX 77840, USA

## Abstract

Genome assembly in its two decades of history has produced significant research, in terms of both biotechnology and computational biology. This contribution delineates sequencing platforms and their characteristics, examines key steps involved in filtering and processing raw data, explains assembly frameworks, and discusses quality statistics for the assessment of the assembled sequence. Furthermore, the paper explores recent Ubuntu-based software environments oriented towards genome assembly as well as some avenues for future research.

## 1. Introduction

Genome assembly involves taking smaller fragments, called “reads,” and assembling them together to form a cohesive unit, called the “sequence.” However, simply assembling all the reads into one contiguous sequence, a “contig,” is not enough. One has to ensure that the assembled sequence does indeed resemble what is truly present in the cell. Some common hurdles are low coverage areas, false positive read-read alignments, false negative alignments, poor sequence quality, polymorphism, and repeated regions of the genome. An even more fundamental concern lies in the difficulty of determining which of the two strands was finally reported in the sequencing procedure. Moreover, as a number of research domains draw suitable conclusions from the sequence itself, a sequence that has not been reported accurately may potentially affect subsequent analyses [1].

Sanger's deoxydinucleotide sequencing with large and accurate reads opened the door to whole-genome sequencing and deciphered the first human genome in 2001 [2, 3]. Sanger's approach is still commercially available with improved capillary electrophoresis, enhanced speed and accuracy, and longer read lengths. NIH's $1,000 genome project led researchers to develop efficient, economical, and high-throughput sequencing platforms introducing a new paradigm called next-generation sequencing (NGS). For instance, Roche's 454 GS, Illumina's MiSeq and HiSeq, ABI's SOLiD, and Life Technologies' Ion Torrent and Proton Torrent platforms all sequence the same genome at a fraction of the time and cost of the first-generation sequencing methods [4].

NGS platforms now produce terabytes of data thereby challenging traditional software tools and hardware architectures which were not designed to process such large amounts of data. This triggered a need to develop algorithms and statistical tools with improved memory management and time complexity in parallel to the development of NGS platforms.

This contribution is intended to act as an introductory note to scientists and researchers working in the area of genome assembly.  Section 2 provides an overview of NGS platforms.  Section 3 discusses raw data, Sequencing Read Archive, and FASTA and FASTQ file formats. It provides particulars on filtering and correcting raw data. Additionally, the second section enforces the need to report accurate results.  Section 4 supplies necessary answers addressing the draft assembly process.  Section 5 reviews common metrics employed to evaluate the assembly and Section 6 highlights recent software environments oriented towards NGS. Finally, Section 7 projects considerations on possible future research trends.

## 2. Overview of Next-Generation Sequencing Platforms

Among NGS platforms, Roche's 454 sequencing is based on Nyren's pyrosequencing approach [5]. Roche's approach, referred to as “sequencing by synthesis” (SS), takes one DNA strand as a template and then uses it to synthesize the sequence of its complementary strand. Roche's SS uses four polymerase enzymes to extend several DNA strands in parallel. Whenever a nucleotide attaches itself onto template DNA, a pyrophosphate molecule is produced which emits light when triggered [6]. The bioluminescence produced by these bases helps in recognizing the bases and, therefore, the sequence. Some characteristics of Roche sequencing include its automated procedures and high speed, while some drawbacks are lower read accuracy for homopolymer segments of identical bases and relatively high operating costs [7].

Illumina, another NGS company, differs from Sanger in several features. Sanger's approach uses dideoxynucleotide for irreversible termination of primer extension, whereas Illumina employs reversible terminators for primer extension of the complementary strand. Illumina's 3-O-azidomethyl reversible terminators are tagged with four different colored fluorophores to distinguish between the four nucleotides. Therefore, using these reversible terminators aids in observing the identity of the nucleotides as they attach onto the DNA fragment because the fluorophores are detected by highly sensitive CCD cameras [8]. Illumina's method significantly reduces the duration of sequencing and assumes a $1000 price tag for 30× human genome. Illumina's sequencing scheme shows some benefits over Roche's pyrosequencing; however, its characteristic short read lengths (<300 bp) present challenges when resolving short sequence repeats.

In addition to Roche and Illumina, Applied Biosystems' SOLiD sequencer is another key player among genome sequencers. SOLiD uses the principle of “sequencing by ligation” (SL). SL differs from Illumina in its method for ligation of octamer oligonucleotides. SL uses dibase fluorescent labeled octaoligonucleotide adaptors which link the template DNA and are bound with 1 *µ*m magnetic beads [9]. At each step, SOLiD's technique encrypts two bases simultaneously and every nucleotide is cross-examined twice: first as the right nucleotide of a pair and then as the left one. This approach reduces homopolymeric sequencing errors. However, similar to Illumina, SOLiD generates short read length data which incur complications in the sequence assembly.

Collectively, these high-throughput sequencers have substantially reduced the cost (≤$0.1/Mb) and duration of genome sequencing. However, additional technologies with enhanced performance have been proposed recently. The advent of nonoptic, semiconductor-based genome sequencers has shown potential. Manufacturers like Life Technologies developed Ion Proton and Ion PGM, both of which use SS amplification and hydrogen ion sensing semiconductors [10]. The sequence is obtained by sensing hydrogen ions emitted when nucleotides incorporate themselves onto template DNA, a process catalyzed by DNA polymerase. Massively parallel transistor-based integrated circuits with about two million wells allow simultaneous detection of multiple reactions. Furthermore, signal processing tools translate voltage fluctuations into base calls for successive nucleotides [10].

Another technique which has been recently proposed is the single-molecule real-time (SMRT) sequencing, introduced by HeliScope [11]. SMRT sequencing scheme is free of library preparation or amplification errors. PacBio RS II (by HeliScope) utilizes SMRT sequencing and can produce about 50,000 reads ranging from 15,000 to 40,000 bases in length in just three hours. The extended read length facilitates sequence alignment and improves precision in drafting an assembly, simply because long repetitive DNA fragments can be easily spanned. Interestingly, Roche will be phasing out its in-house 454 sequencers in 2016 in favor of PacBio's SMRT sequencers. Roche plans to maintain its participation in NGS market, not by developing its own sequencers, but rather by becoming an exclusive seller for in vitro diagnostic products based on PacBio's SMRT sequencing platform (http://www.bio-itworld.com/BioIT_Article.aspx?id=131053, accessed on Dec. 12, 2015). Together with nonoptic semiconductor nanopore technology, SMRT sequencers are referred to as “third-generation-sequencers” [12–14]. Overall, the above-mentioned high-throughput sequencers have substantially reduced the duration and cost of sequencing ($0.1/Mb).

Companies are investing significant resources to upgrade existing technologies and introduce newer machines. It is hoped that many third-generation-sequencers are expected to surface, coupling SMRT sequencing with principles of electrothermodynamics, quantum physics, and nanopore technology [13–15]. Existing platforms are currently designed to cater for de novo synthesis, wholegenome/whole-exome and transcriptome synthesis, targeted resequencing, RNA profile ChIP-Seq, mutation detection, and metagenomics. Platforms are usually accompanied by bioinformatics tools. Tables 1, 2, and 3 present some important details about current sequencers.

## 3. Preliminary Data Processing Steps

**Figure 1:**
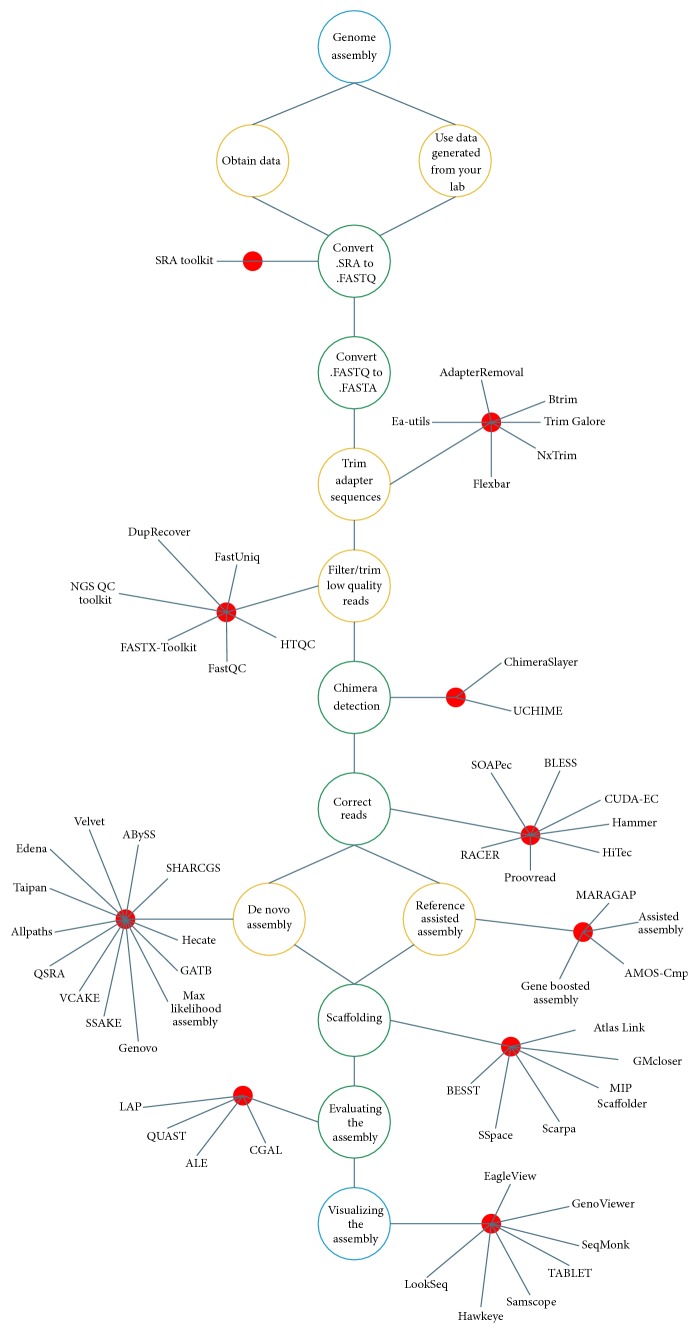
Flow chart for DNA assembly pipeline. Some commonly used tools are mentioned next to each step [36]. Please refer to [19, 35, 37–88] for details on the above-mentioned tools.

Software tools and applications enter the research process once the sequencers fulfill their role of generating reads. The aim of this and the next set of sections is to provide an outline of the individual steps involved in transforming raw data into the novel genome, as presented in Figure 1. The set of interconnected methods are referred to as a “pipeline.” The process starts by using the data generated by one's lab or by downloading the data from the Sequencing Read Archive (SRA) [16]. Data is present in “.SRA” format and must be converted into  .FASTQ file format by employing the SRA toolkit (http://www.ncbi.nlm.nih.gov/Traces/sra/). Once converted, the FASTQ format adopts a four-line representation to display the sequence and its associated quality [1]:

 
*@ Sequence Identifier*
 
*Sequence line(s)*
 
*+ Sequence Identifier*
 
*ASCII encoding of quality values*

ASCII characters utilized in the last line of the above-mentioned SRA format symbolize quality values (*Q*-values). *Q*-values are log-probabilities illustrating the quality of each base call. For example, for Sanger the formula is

(1) $$\begin{array}{c} Q_{PHRED}=-10\times {log}_{10}\left(P_{e}\right), \end{array}$$

where *P*_*e*_ is the probability of determining a base incorrectly [17, 18]. For ASCII encoded quality values the following characters depict an increasing order of quality:

 !*”*#$%&*'*()*∗* + , −./0123456789:; < = >?@ABCDEFG
 HIJKLMNOPQRSTUVWXYZ[∖^∧^ *‘*abcdefghijklmno
 pqrstuvwxyz{∣}~

Similar to FASTQ, FASTA format seems like an abridged version of FASTQ file format. It maintains a two-line arrangement to display the sequence and contains no mention of its quality:

 
*@ Sequence Identifier*
 
*sequence line(s)*

Once reads are received in their correct format, one must trim adapter sequences, filter, or trim low quality ends and collapse identical reads. A naive approach is to remove all reads that contain the flag “*N*.” An improved method retains all reads that have an overall quality *P*_qual_ > *q*, where *q* is a user-defined parameter [19–23]. A more enhanced approach consists in matching reads against known ribosomal and heterochromatin DNA and removing them should they match [24]. Nevertheless, since a significant portion of raw data contains errors one must correct them.

## 4. Assembly Process

**Figure 2:**
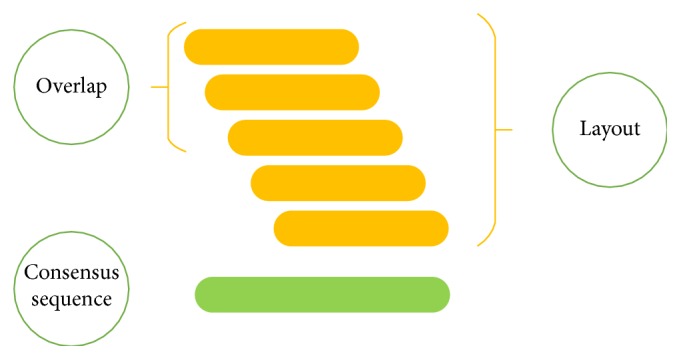
De novo assembly: reads that* overlap* each other are shown to align at appropriate places with respect to one another, thereby generating the* layout*. The layout, in turn, constructs a* consensus* sequence, simply by basing itself on the majority base call. The above-mentioned framework is called “*Overlap-Layout-Consensus*.”

**Figure 3:**
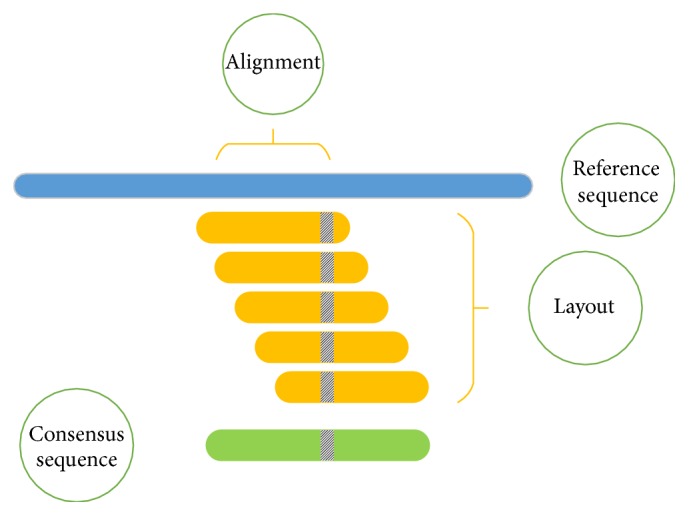
*Reference assisted assembly*: reads* align* relative to a reference sequence setting up the* layout*. The layout, in turn, constructs a* consensus* sequence, simply by basing itself on the majority base call. Please note that the reads do not need to match perfectly with the reference. The example shows a shaded region where the consensus sequence differs from the reference. This working scheme is called “*Alignment-Layout-Consensus*.”

The primary aim of the assembly process is to connect all reads together, one after another, to form a single contiguous sequence. Interestingly, due to the inherent nature of the problem, graph theory, especially de Bruijn graph, models very well such a process [25]. In graphical models individual nodes symbolize reads whereas edges between the nodes emphasize “overlaps” between reads. Once the overlap between all reads is established, the task at hand is to generate a “layout” by searching for a single path from beginning, that is, the root of the graph structure, to the end, the leaf of the graph structure, as illustrated in Figures 2 and 3. As such, generating a layout is very challenging, because not one but multiple disjointed graphs are realized, each depicting a contig. In addition, each graph has many loops portraying repeat regions as well as multiple branches, both long and short. All these hazards need to be resolved. Branches that are small may be discarded, while longer branches compete with one another to serve as potential representatives for each contig. Loops portray repeat regions, so one must decide how many times the repeats should be placed within the final assembly. Nevertheless, assemblers do spend a significant amount of time resolving potential hazards, in multiple ways. The output is a collection of contigs that need to be ordered, appended, and elongated, a process called “scaffolding” [25–28].

## 5. Evaluating the Quality of an Assembly

Evaluating the quality of an assembly requires analyzing multiple metrics. These statistics measure an assembly from various standpoints.  Table 4 illustrates some commonly used assembly metrics/statistics and their explanations. After evaluating the assembly it is recommended to visualize the assembly in order to obtain a pictorial view of the draft. Figure 1 presents common tools used in each part of the pipeline.

## 6. Linux Based Distributions

The software environments pertaining to genome assembly are many and as such need to be constantly maintained, configured, and updated. This repeated and continuous configuration consumes a good amount of time and resources. Therefore, to address these challenges, engineers and computer scientists have proposed multiple solutions built on Linux systems that include within them all the necessary software needed by the research group.  Table 5 mentions a few. As for genome assembly, both Baari, an Ubuntu-derived operating system (http://people.tamu.edu/~bilalwajidabbas/Baari.html), and Genobuntu, a software package, provide about 60+ genome assembly tools (https://sourceforge.net/projects/genobuntu/). It is hoped the current set of tools will be constantly updated to suit the ever growing needs of the scientific community.

## 7. Considerations and Concerns

Genome Online Database (GOLD) reports that as of Dec 12, 2015, 1,136 Archaeal, 49,983 Bacterial, 4,473 Viruses, and 11,122 Eukaryotic genomes have been sequenced. There remains plenty of room for work. The $1000 genome project has reduced the cost significantly, but if personalized medication is expected to be effective and available to everyone, the cost and time duration for sequencing need to be reduced further. Processing raw data needs to be done both cheaply and at ultra-fast rates. Spending about 50 hours of processing time on a system with 20 microprocessor cores and 20 GB RAM is not uncommon (as of 2014) [29]. Imagine trying to sequence the genomes of an entire country's population. Transferring all the raw data via an Internet connection from one country to another is not feasible. Therefore, countries will have to provide for their own supercomputers, and algorithms will need to be parallelized with careful attention to Hadoop and MapReduce frameworks [30–34]. Hadoop and MapReduce are ideal as both are designed to process “big-data” using parallel and distributed algorithms on clusters of systems [30–34]. With so many obstacles ahead, genome assembly will remain challenging for many years to come.

## Figures and Tables

**Table 1:** Comparison of current (as of Nov. 15, 2014) sequencing platforms. PCR: polymerase chain reaction, SS: sequencing by synthesis, SL: sequencing by ligation, SH: sequencing by hybridization, and SE: sequencing by expansion.

Platform	Biochemistry/ biotechnology	Amplification	Throughput	Reads per run	Read length (bp)	Seq run time	Error rate (%)	Machine cost ×1000	Cost per run	Cost per unit data
Sanger (Applied Biosystems 3730xl)	Dideoxynucleotide termination of PCR	PCR	0.06 Mb	9600	1000	2 hrs	0.1	$100	$100	$8,000–$10,000/Gb

454 GS+	Bioluminescence on nucleotide incorporation	Emulsion PCR	~70 Mb	70 k~100 k	~700	18 hours	<1.0	$125	$1,000	$28.50/Gb

454 GS FLX+	Bioluminescence on nucleotide incorporation	Emulsion PCR	700 Mb	1 M	~1000	23 hours	<1.0	$500	$6,000	$8.50/Gb

MiSeq	Cleavage of 3′-O-azidomethyl reversible terminator and fluorescent tag on nucleotide incorporation	SS	15 Gb	25 M	2W300	5~55 hrs	0.1	$125	$1.4K	$93/Gb

HiSeq X Ten	Cleavage of 3′-O-azidomethyl reversible terminator and fluorescent tag on nucleotide incorporation	SS	1000 Gb	4000 M	2W125	7 hrs~6 d	0.1	$1,000	$12K	$7/Gb

NextSeq 500	Cleavage of 3′-O-azidomethyl reversible terminator and fluorescent tag on nucleotide incorporation	SS	129 Gb	400 M	2W150	26~29 hrs	0.1	$250	$4K	$33/Gb

SOLiD 5500xl	Ligation of octamer oligonucleotide and cleavage of fluorescent tag	SL	180 Gb	2.8 B	2W60	150 hrs	0.01	$595	$10K	$9/Gb

Ion Proton I	Proton sensing by pH change	SS	10 Gb	40~80 M	200	2~4 hrs	1.0	$149	$1K	$100/Gb

Ion PGM 318	Proton sensing by pH change	SS	2 Gb	5 M	400	7.3 hrs	1.0	$52	$750	$350/Gb

Polonator G.007	Cleavage of 3′-ONH2 reversible terminator and fluorescent tag on nucleotide incorporation	SL	10 Gb	—	26	—	N.A	N.A	N.A	N.A

Helicos HeliScope	Single-molecule real-time sequencing	SS	35 Gb	20 M	35	8 hrs	0.5	$1,000	$10K	$330/Gb

PacBio RS II	Single-molecule real-time sequencing	SS	1 Gb	50,000	15,000 bp	3 hrs	15	$700	$400	~$1000/Gb

**Table 2:** The table enlists the strong points and challenges pertaining to some of the sequencing platforms.

Platform	Positive points	Challenges
Sanger (Applied Biosystems 3730xl)	Long read length; good for individual gene analysis	Slow; expensive; poor quality due to primer dimer

454 GS+	Long read length; fast; low cost for small studies	High error rate for homopolymer read; low throughput; will be phased out in 2016

454 GS FLX+	Long read length	High error rate homopolymer read; low throughput; large capital cost; will be phased out in 2016

MiSeq	High throughput; ideal for small genome project	Short read length

HiSeq X Ten	High throughput; ideal for whole-genome project	Short read length

NextSeq 500	High throughput; ideal for small to large scale project	Short read length

SOLiD 5500xl	High throughput	Short read length; poor output data distribution and arduous data analysis

Ion Proton I	Ideal for small project; shorter run time; leading future technology	Higher error rate; larger cost per Mb

Ion PGM 318	Low capital investment and running cost; shorter run time	Higher error rate; larger cost per Mb

Polonator G.007	Cost-effective; open resource	Obsolete

Helicos HeliScope	Single-molecule sequencing; simple sample preparation and data analysis	Short read length; obsolete

PacBio RS II	Single-molecule real-time sequencing; longest available read length	High error rate

**Table 3:** Recent sequencing platforms: these platforms are relatively new and to date (Nov. 15, 2014) there is not enough information to incorporate them into Table 1.

Platform	Company	Biotechnology	Resource
GENIUS	GenapSys	Proton sensing by pH and temperature change	http://genapsys.com/

NanoTag sequencer	Genia	Electric current change produced by nanotag released from incorporation of nucleotide	http://geniachip.com/

GnuBIO platform	GnuBIO system	Oligo hexamers hybridization in microfluidics	http://gnubio.com/

*∗*	Lasergene	3′-OH unblocked reversible terminator	http://lasergen.com/

*∗*	Nabsys	Hexamer oligonucleotides hybridization mapping through nanopore arrays	http://nabsys.com/

MinION and GridION	Oxford Nanopore Technologies	Strand DNA or exonuclease cleaved nucleotides pass through nanopores change electric current flow rate	https://nanoporetech.com/

*∗*	Strato Genomics Technology	Conversion of DNA into Xpandomer	http://stratosgenomics.com/

^*∗*^Lasergene, Nabsys, and Strato Genomics are working on newer platforms.

**Table 4:** Some common assembly statistics. Here an ↑ indicates higher is better while a ↓ implies less is better.

↑/↓	Description
↑	*N50*: quantified the length of the scaffold at which 50% of the total assembled size of the sequence is covered. *NG50*: evaluated in a way similar to N50. However, here the length of the sequence is either known or predicted [1, 29]. *NA50* and *NGA50*: these metrics deal with aligned blocks rather than contigs [35]. *Continuity*: similar to N50, NA50, NG50, and NGA50 there are other metrics like *N75, NA75, NG75, NGA75, N90, NA90, NG90*, and *NGA90*. *Number of Genes*: an assembly which exhibits more highly conserved core Eukaryotic genes is considered better [29]. *Accuracy*: if an assembly reports at least 90% of its bases with a minimum of 5× coverage, it is considered accurate. *Choppiness*: the average contig length should be greater than a certain threshold. Otherwise, the assembly needs to be redrafted. *Validity*: the fraction of assembly that can be confirmed by a reference sequence [29]. *Completeness*: an assembly is considered complete if the scaffolds cover more than 90% of the actual genome. *Length of the Longest Scaffold*: typically the greater the length, the better the assembly. Similar is the case of the *shortest scaffold*. *Number of scaffolds* > *X*, where *X* is a user-defined length. Similarly, *% age of scaffolds* > *X*. *Total Length of the Scaffolds* and *Total Scaffold Length as Percentage of Estimated Genome Size*: the closer it is to 100%, the better it is. *Percentage of Contigs Scaffolded*: percentage of contigs that were connected with one another during the scaffolding process [1].

↓	*Number of Gaps in the Assembly*: by aligning paired-read data onto scaffolds one may determine scaffolding errors [1]. *Number of Scaffolds*: an assembly which has a smaller number of scaffolds would be assumed to be better. For example, the optimum assembly would be one continuous sequence depicting the true sequence. *LG50 Scaffold Count*: number of scaffolds counted in reaching NG50 threshold. Similar would be the case of *LG75* and *LG90*. *Percentage of Unscaffolded Contigs*: since contigs may remain unscaffolded.

**Table 5:** Comparison of different Linux distributions. Here LTS stands for Long Term Support and GUI refers to Graphical User Interface.

Operating system	Free	Base OS	Software	Open source	LTS	GUI	×86/×64	Cloud	Script files
Baari	✓	Ubuntu 13.10	60+ genome assembly tools	✓	✓	Unity	×64	×	✓

Lxtoo	✓	Gentoo Linux 11	Sequence analysis, protein-protein interactions	✓	✓	X11 Desktop	×86/×64	×	×

Open Discovery 3	×	Fedora Sulphur 9	Molecular dynamics, docking, sequence analysis	×	✓	GNOME 2.22	×86/×64	✓	×

BioBrew	✓	Red Hat 7.3	Appropriate for clusters	✓	×	KDE, GNOME	×86	×	×

PhyLIS	✓	Ubuntu 8	Phylogenetics	✓	×	Unity	×86/×64	×	×

DNALinux	✓	Xubuntu	DNA and protein analysis	✓	×	XFCE 4.2.2	×86	✓	×

Bioconductor Buntu	✓	Ubuntu 12.04	Bioconductor Buntu 2.11	✓	✓	Unity	×86/×64	×	×

BioLinux 7	✓	Ubuntu 12.04	500+ bioinformatics applications with 7 assembly tools	✓	✓	Unity	×64	✓	×

## Key Points

- NIH's $1,000 genome project led researchers to develop efficient, economical, and high-throughput sequencing platforms. Examples include Roche's 454 GS, Illumina's MiSeq and HiSeq, ABI's SOLiD, and Life Technologies' Ion Torrent and Proton Torrent platforms. A brief comparison of these next-generation sequencing platforms is presented.
- Data provided by these platforms is transformed into a sequence via a series of processes collectively called a “pipeline.” It starts with trimming adapter sequences, filtering low quality ends, and collapsing identical reads. The final set of reads are then connected together, one after another, to form contiguous sequences, called “contigs.” The collection of contigs needs to be ordered, appended, and elongated via a process called “scaffolding.”
- A number of software environments providing bioinformatics solutions have been provided over the years. A brief comparison of some of these is presented here.
